# Knowledge mapping of ferroptosis in Parkinson’s disease: a bibliometric analysis: 2012–2023

**DOI:** 10.3389/fnagi.2024.1433325

**Published:** 2024-08-30

**Authors:** Juanqin Li, Yanli Wang, Jing Huang, Daokai Gong

**Affiliations:** ^1^Department of Neurology, Jingzhou Hospital Affiliated to Yangtze University, Jingzhou, China; ^2^Hubei Provincial Clinical Research Center for Parkinson’s Disease, Xiangyang, China

**Keywords:** ferroptosis, Parkinson’s disease, RStudio bibliometrix, CiteSpace, VOSviewers, bibliometrics

## Abstract

**Background:**

Ferroptosis is a crucial pathogenic mechanism in Parkinson’s disease, offering significant potential for pharmacological intervention. Despite its importance, the number of bibliometric analyses examining the relationship between ferroptosis and Parkinson’s disease remains limited. This study aims to elucidate the knowledge structure and primary research focuses within this field using various bibliometric tools search.

**Materials and methods:**

We conducted a comprehensive literature son ferroptosis in Parkinson’s disease using the Web of Science Core Collection database. Bibliometric analyses and visualizations were performed with VOSviewer, examining the geographical and institutional distribution of publications, journal interconnections, and keyword prevalence. Furthermore, CiteSpace was used to visually explore and analyze journal interactions and citation dynamics. The bibliometrix R package facilitated the delineation of collaborative networks across different countries and the construction of visual network representations illustrating relationships among authors, keywords, and journals. Data visualization was further enhanced with Microsoft Office Excel 2021.

**Results:**

Recently, there has been a significant increase in publications on ferroptosis, with China emerging as a leading contributor in this research area. Keyword analysis highlights the critical role of ferroptosis in the pathogenesis of Parkinson’s disease, identifying GPX4 as a key enzyme mitigating lipid peroxidation. This study also elucidates the connections and distinctions between ferroptosis and other cell death processes such as apoptosis, autophagy, and pyroptosis. Current research primarily focuses on immunotherapy, prognosis, oxidative stress, lipid peroxidation, and the tumor microenvironment.

**Conclusion:**

This study provides a comprehensive initial analysis of the research landscape, identifying current focal points and potential future directions for ferroptosis research in Parkinson’s disease. The findings leverage a variety of bibliometric methodologies to offer valuable insights into this emerging field.

## 1 Introduction

Parkinson’s disease (PD) is the second most prevalent neurodegenerative disorder globally, after Alzheimer’s disease. The incidence of PD is rising, driven by an aging population, particularly in China, and a concerning trend toward earlier onset in younger individuals (Dorsey et al., 2018; Pringsheim et al., 2014). Consequently, research focused on the prevention and management of PD has become increasingly vital. Despite significant advances, the etiology of PD remains largely unclear, with current treatments predominantly addressing symptoms rather than underlying causes (Armstrong and Okun, 2020; Bloem et al., 2021). Recent studies have highlighted ferroptosis as a key pathogenic mechanism in PD, positioning it as a promising target for therapeutic interventions (Do Van et al., 2016; Guiney et al., 2017; Jiang et al., 2023).

Ferroptosis, identified by Dixon et al. (2012), is a distinct form of cell death that differs from apoptosis, necrosis, and autophagy in its morphology, mechanism, and molecular pathways. It is characterized by iron-dependent lipid peroxidation, which damages the cell membrane and leads to cell death (Mortensen et al., 2023). This process can be mitigated by iron chelators or antioxidants like glutathione peroxidase 4 (GPX4). The regulation of ferroptosis involves a complex interplay of mechanisms, including iron chelation, antioxidant defense, and lipid repair (Stockwell, 2022). Ferroptosis is involved in various diseases, including neurodegenerative disorders, cancer, and ischemia-reperfusion injuries (Jiang et al., 2021; Stockwell, 2022; Stockwell et al., 2017). Thus, targeting the ferroptosis pathway presents a promising approach for therapeutic development and disease management.

Bibliometrics is a systematic methodology for evaluating and analyzing literature through quantitative measures, using mathematical and statistical techniques (Liu et al., 2024; Sun et al., 2023; Zhang et al., 2022). This approach provides insights into publication volumes, author and institution productivity, citation patterns, and the interdisciplinary evolution of research fields. Advanced visual analytics tools facilitate the identification of leading journals, research entities, influential authors, and emerging research trends (Donthu et al., 2021; Pan et al., 2018). Knowledge graphs, generated using software such as VOSviewer (Thomas and Lawrence, 2022; van Eck and Waltman, 2010), CiteSpace (Chen et al., 2012), and the R package bibliometrix (Kemeç and Altınay, 2023), offer a detailed overview of research landscapes. Databases like Web of Science, Scopus, and Google Scholar provide comprehensive bibliometric analysis capabilities, supporting thorough and nuanced research investigations (McNicholas et al., 2022).

## 2 Materials and methods

### 2.1 Data sources and retrieval strategies

On March 22, 2024, we conducted a systematic literature search using the Web of Science Core Collection (WoSCC) database. Renowned for its comprehensive and high-quality collection of scholarly literature, WoSCC is considered the gold standard for bibliometric analysis (Ding and Yang, 2020; Ma et al., 2021; Merigó and Yang, 2017). Our search utilized the terms “ferroptosis” and “Parkinson’s disease,” structured as TS = (ferroptosis OR ferroptotic OR “iron death”) AND TS = (Parkinson* OR PD). Since “ferroptosis” was first introduced in 2012, the search was limited to the period from January 1, 2012, to December 31, 2023. This search strategy identified 510 documents. After filtering to include only “articles” and “review articles” in English, the dataset was refined to 495 documents, comprising 347 articles and 148 review articles.

### 2.2 Data analysis

To perform the bibliometric analysis, we utilized VOSviewer, CiteSpace, and bibliometrix R package three widely recognized tools for quantitative literature assessment. VOSviewer version 1.6.19.0 was used to analyze the distribution of publications by country, institution, and journal, as well as to assess co-citation patterns and keyword frequencies. The VOSviewer analysis parameters included: counting method (full counting), visualization weights for countries, institutions, journals, and author keywords (documents), visualization weights of co-cited journals (citations), visualization weights of author keywords (occurrences), normalization method (association strength), clustering resolution (1.00), minimum cluster size (1), minimum strength (0), and maximum lines (1,000).

CiteSpace version 6.1.R6, developed in Java, facilitated the visualization and analysis of journal and reference data. Following the import of the literature into CiteSpace, the data were formatted according to the software’s requirements. The analysis parameters set included: time span (January 2012–December 2023), time slicing (1 year), *g*-index (*k* = 25), node types (Reference), top *N* (50), and clustering method (LLR), with other settings at default. This setup enabled the generation of visualizations for journal and reference networks.

The bibliometrix R package 4.1.3, used with RStudio 2023.03.1 and R version 4.3.1, was utilized for mapping collaborative networks between countries and constructing visual network views for authors, keywords, and the interconnections among authors, keywords, and journals. For analyzing trending topics in authors’ keywords, the parameters included: time span (2013–2023), word minimum frequency (5), number of words per year (3), with other settings at default. For general bibliometric data—such as number of publications (NPs), countries, institutions, journals, authors, and citation frequency—graphs were created using Microsoft Office Excel version 2021.

## 3 Results

### 3.1 Basic quantitative information

The defined search strategy yielded 495 documents authored by 3,269 individuals from 693 institutions across 47 countries. These documents were published in 243 journals, accumulating a total of 30,206 citations from 3,253 distinct journals.

### 3.2 Quantitative analysis of publications

**Figure 1:**
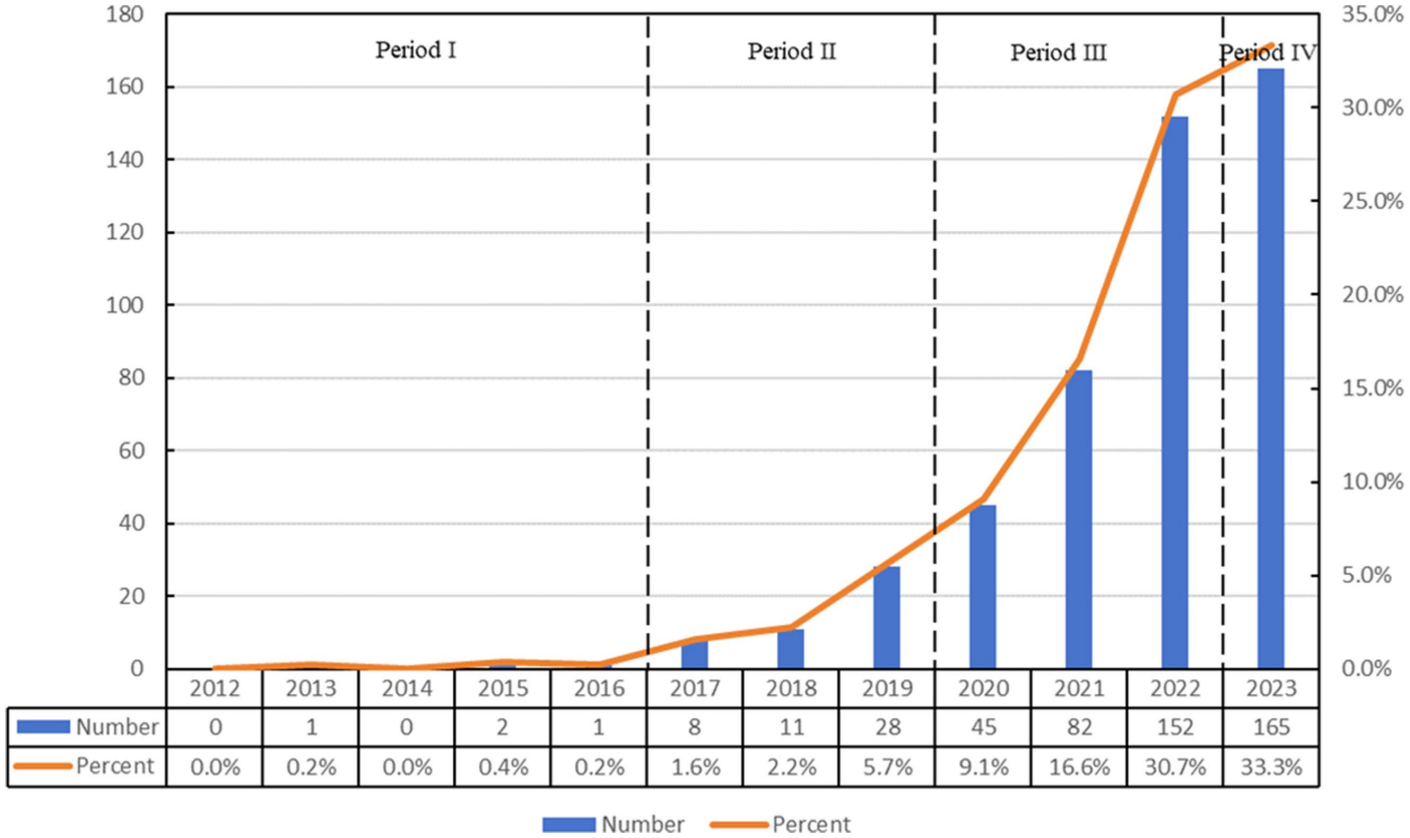
Annual output of research on ferroptosis in Parkinson’s disease.

Over the past 11 years (2012–2023), our search identified 495 articles from the Web of Science database that examine the relationship between ferroptosis and Parkinson’s disease. This collection comprises 347 research articles and 148 review articles. The temporal distribution of these publications, as illustrated in Figure 1, demonstrates a consistent increase in research activity, with a significant surge starting in 2017.

The evolution of publication volume over this period can be divided into four separate phases: the initial phase (2012–2016), the early development phase (2017–2019), the rapid growth phase (2020–2022), and the current phase (2023 to present). During the initial phase, there were only four publications, reflecting nascent interest in the relationship between ferroptosis and Parkinson’s disease. After 2016, the annual research output began to increase. The early development phase is marked by an average annual output of approximately 15 publications, indicating a preliminary exploration stage. The rapid growth phase is characterized by a substantial increase in publications. For example, in 2022, the publication count reached 152 articles, which was 1.85 times the output of the preceding year, accounting for a 56.4% growth rate during this stage. Although the current phase continues to show an upward trend, the rate of increase has started to stabilize, with 165 publications recorded in 2023, representing 33.3% of the overall publication count. This trajectory highlights the increasing prominence of the relationship between ferroptosis and Parkinson’s disease as a focal point of future research endeavors.

### 3.3 National and institutional analysis

**Figure 2:**
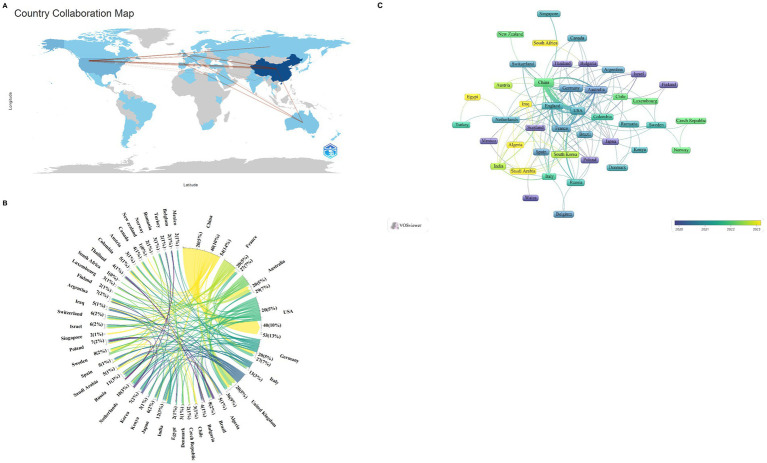
Geographical distribution **(A)**, chord diagram of network relations between countries **(B)**, and national visualization analysis **(C)** of ferroptosis in Parkinson’s disease.

The analyzed publications span 47 countries and 693 distinct institutions, with the top nine countries representing a global distribution across Asia, North America, Europe, and Oceania. Notably, Europe and Asia dominate the publication landscape, as detailed in Table 1. This distribution reflects global patterns of economic, social, and cultural development. Among these nations, China stands out as the leading contributor, with a substantial lead in publication volume (*n* = 349, 66.9%), followed by the United States (*n* = 67, 12.8%), Australia (*n* = 21, 4.0%), and Germany (*n* = 19, 3.6%). These four countries thus form the cornerstone of research into ferroptosis in the context of Parkinson’s disease. For further analysis, we focused on the 47 countries with at least one publication. A collaborative network graph was constructed to illustrate publication output and collaboration dynamics among these nations (Figure 2). In this graph, the thickness of the matrix in the labeled view represents the volume of literature published by each country in this field, while the lines between nodes indicate the strength of collaboration. Thicker lines denote more frequent collaborative efforts. The color gradient from cold to warm represents the chronological proximity of the collaboration, with warmer colors indicating more recent interactions. For example, China has close collaborations with the United States, Australia, Germany, and the United Kingdom, while the United States also actively collaborates with China, Germany, and the United Kingdom.

**Table 1:** Countries and institutions leading in ferroptosis research in Parkinson’s disease.

Rank	Countries	Counts	Institutions	Counts
1	China	349 (66.9%)	Chinese Academy of Sciences	18 (13.8%)
2	The United States	67 (12.8%)	Shanghai Jiao Tong University	17 (13.1%)
3	Australia	21 (4.0%)	Fudan University	16 (12.3%)
4	Germany	19 (3.6%)	The University of Melbourne	16 (12.3%)
5	England	17 (3.3%)	Central South University	14 (10.8%)
6	France	15 (2.9%)	Huazhong University of Science and Technology	13 (10.0%)
7	Italy	13 (2.5%)	China Medical University	12 (9.2%)
8	India	12 (2.3%)	Qingdao University	12 (9.2%)
9	Japan	9 (1.7%)	Zhejiang University	12 (9.2%)

**Figure 3:**
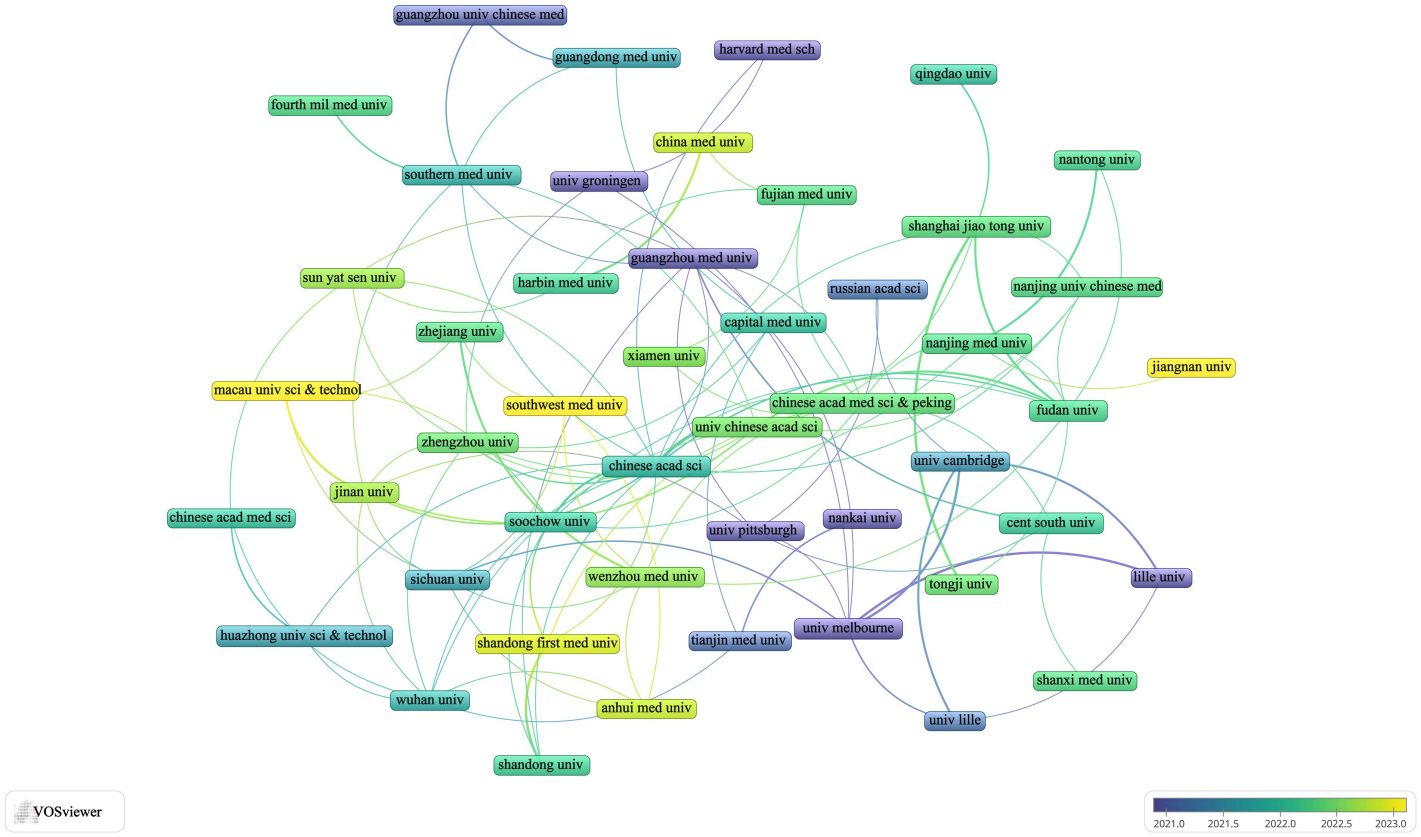
Institutional visualization analysis of ferroptosis in Parkinson’s disease.

Among the top nine institutions engaged in this research area, eight are located in China. The leading institutions in terms of publication volume included the Chinese Academy of Sciences (*n* = 18, 13.8%), Shanghai Jiao Tong University (*n* = 17, 13.1%), Fudan University (*n* = 16, 12.3%), and the University of Melbourne (*n* = 16, 12.3%) (Table 1). For further analysis, we selected 82 of the 693 institutions with a publication volume of at least three articles. A collaborative network view was constructed based on each institution’s publication volume and their interrelationships (Figure 3). In this figure, the thickness of the matrix indicates the volume of publications by each institution, highlighting the Chinese Academy of Sciences as the most prolific entity. The thickness of the lines represents the intensity of cooperation between institutions, with thicker lines indicating a greater number of co-authored papers. For instance, the Chinese Academy of Sciences has close collaborations with the University of Chinese Academy of Sciences, Soochow University, and the First Medical University of Shandong Province. Meanwhile, the University of Melbourne has established significant collaborations with the University of Lille, the University of Cambridge, and Sichuan University. The color gradient from cold to warm in Figure 3 visualizes the temporal proximity of these collaborations, highlighting the core research institutions in this field.

### 3.4 Journals and co-cited journals

The articles analyzed were published across 243 journals focusing on ferroptosis in Parkinson’s disease. Based on Bradford’s law, we identified 19 core journals. Among these, the *International Journal of Molecular Sciences* published the most articles (*n* = 16, 9.7%), followed by *Frontiers in Oncology* (*n* = 14, 8.5%), *Free Radical Biology and Medicine* (*n* = 13, 7.9%), and *Frontiers in Cell and Developmental Biology* (*n* = 13, 7.9%). The *h*-index, introduced by Hirsch (2005), evaluates a journal’s productivity and citation impact. The *h*-index determines the number of articles (*h*) that have been cited at least *h* times each, reflecting the journal’s influence in the field (Hou et al., 2022). As detailed in Table 2, the journal with the highest *h*-index is *Free Radical Biology and Medicine* (*h*-index = 11), followed by *Oxidative Medicine and Cellular Longevity* (*h*-index = 8). Additionally, the *g*-index, proposed by Egghe (2006) and Woeginger (2008), measures both the impact of each paper and the overall impact of the journal’s output. According to Table 2, the highest *g*-index is found in the *International Journal of Molecular Sciences* (*g*-index = 15), followed by *Free Radical Biology and Medicine*, *Frontiers in Cell and Developmental Biology*, and *Oxidative Medicine and Cellular Longevity*. Among these journals, *Free Radical Biology and Medicine* leads in total citations (TC = 1,362), followed by *Frontiers in Neuroscience* (TC = 848) and *Molecular Neurobiology* (TC = 427).

**Table 2:** Core journals in the field of ferroptosis research in Parkinson’s disease.

Rank	SO	Counts	*h*-index	*g*-index	TC
1	International Journal of Molecular Sciences	16 (9.7%)	7	15	250
2	Frontiers in Oncology	14 (8.5%)	4	9	90
3	Free Radical Biology and Medicine	13 (7.9%)	11	13	1,362
4	Frontiers in Cell and Developmental Biology	13 (7.9%)	7	13	169
5	Antioxidants	11 (6.7%)	6	9	93
6	Oxidative Medicine and Cellular Longevity	11 (6.7%)	8	11	398
7	Advanced Science	10 (6.1%)	6	9	90
8	Frontiers in Genetics	10 (6.1%)	5	7	56
9	Frontiers in Immunology	10 (6.1%)	5	10	148
10	Ageing Research Reviews	7 (4.2%)	5	7	163
11	Molecular Neurobiology	7 (4.2%)	4	7	427
12	Cancer Cell International	6 (3.6%)	4	6	46
13	Cells	6 (3.6%)	4	6	43
14	Frontiers in Pharmacology	6 (3.6%)	4	6	164
15	Biomedicines	5 (3.0%)	3	5	45
16	Cell Death & Disease	5 (3.0%)	4	5	177
17	Frontiers in Molecular Biosciences	5 (3.0%)	4	4	24
18	Frontiers in Neuroscience	5 (3.0%)	5	5	848
19	Neural Regeneration Research	5 (3.0%)	5	5	237

**Figure 4:**
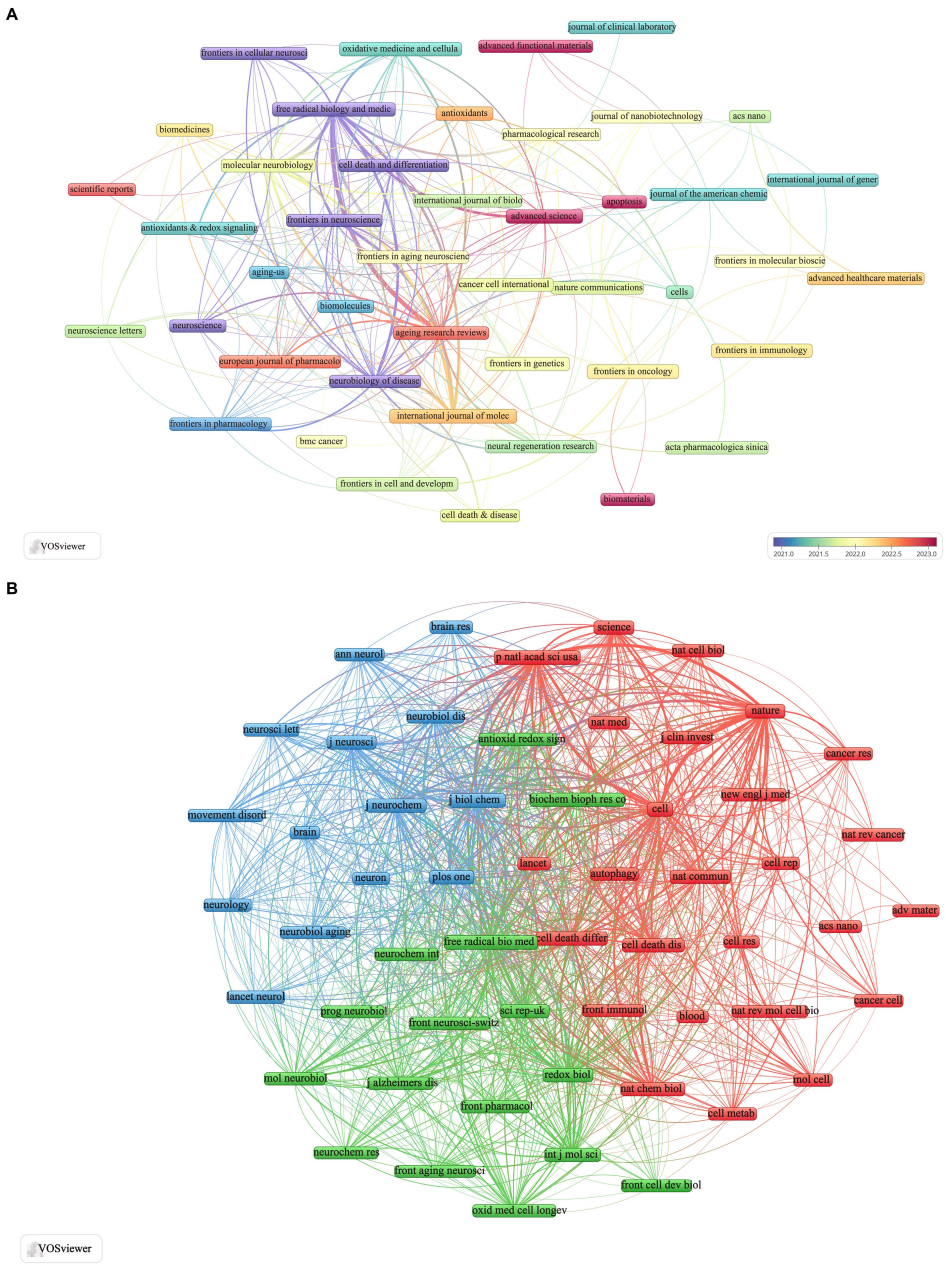
Visualization of ferroptosis in Parkinson’s disease in journals **(A)** and co-cited journals **(B)**.

For a more detailed analysis, we screened 44 journals with three or more relevant publications and created a network view of these journals (Figure 4A). This figure illustrates that the *International Journal of Molecular Sciences* has strong collaborations with *Frontiers in Neuroscience*, *Molecular Neurobiology*, *Free Radical Biology and Medicine*, and *Neurobiology of Disease*. Additionally, *Free Radical Biology and Medicine* maintains close relationships with journals such as *Ageing Research Reviews*, *International Journal of Molecular Sciences*, *Molecular Neurobiology*, and *Frontiers in Aging Neuroscience*.

Table 3 lists 11 journals that have each accrued more than 500 TCs. Notably, *Cell* and *Nature* lead with co-citations totaling 1,269 and 1,200, respectively. *Nature* has the highest impact factor (2022–2023) at 64.8, closely followed by *Cell* with an impact factor of 64.5. From an initial pool of 3,253 journals, we selected 56 with 167 or more co-citations and visualized their network (Figure 4B). The clustering view in Figure 4B reveals strong relationships between *Nature*, *Cell*, *Proceedings of the National Academy of Sciences of the United States of America*, *Journal of Biological Chemistry*, and *Free Radical Biology and Medicine*. This inter-journal co-citation highlights the central role these publications play in disseminating influential research findings on ferroptosis in Parkinson’s disease, underscoring their authoritative position in the field.

**Table 3:** Top 11 co-cited journals ferroptosis in Parkinson’s disease research.

Rank	Co-cited journal	Co-citations	IF (2022)	JCR
1	Cell	1,269	64.5	Q1
2	Nature	1,200	64.8	Q1
3	Journal of Biological Chemistry	895	4.8	Q2
4	Proceedings of the National Academy of Sciences of the United States of America	876	11.1	Q1
5	Free Radical Biology and Medicine	842	7.4	Q1
6	Journal of Neurochemistry	620	4.7	Q2
7	Cell Death and Differentiation	588	12.4	Q1
8	Nature Communications	582	16.6	Q1
9	Redox Biology	565	11.4	Q1
10	Cell Death & Disease	553	9.0	Q1
11	PLoS One	506	3.7	Q2

**Figure 5:**
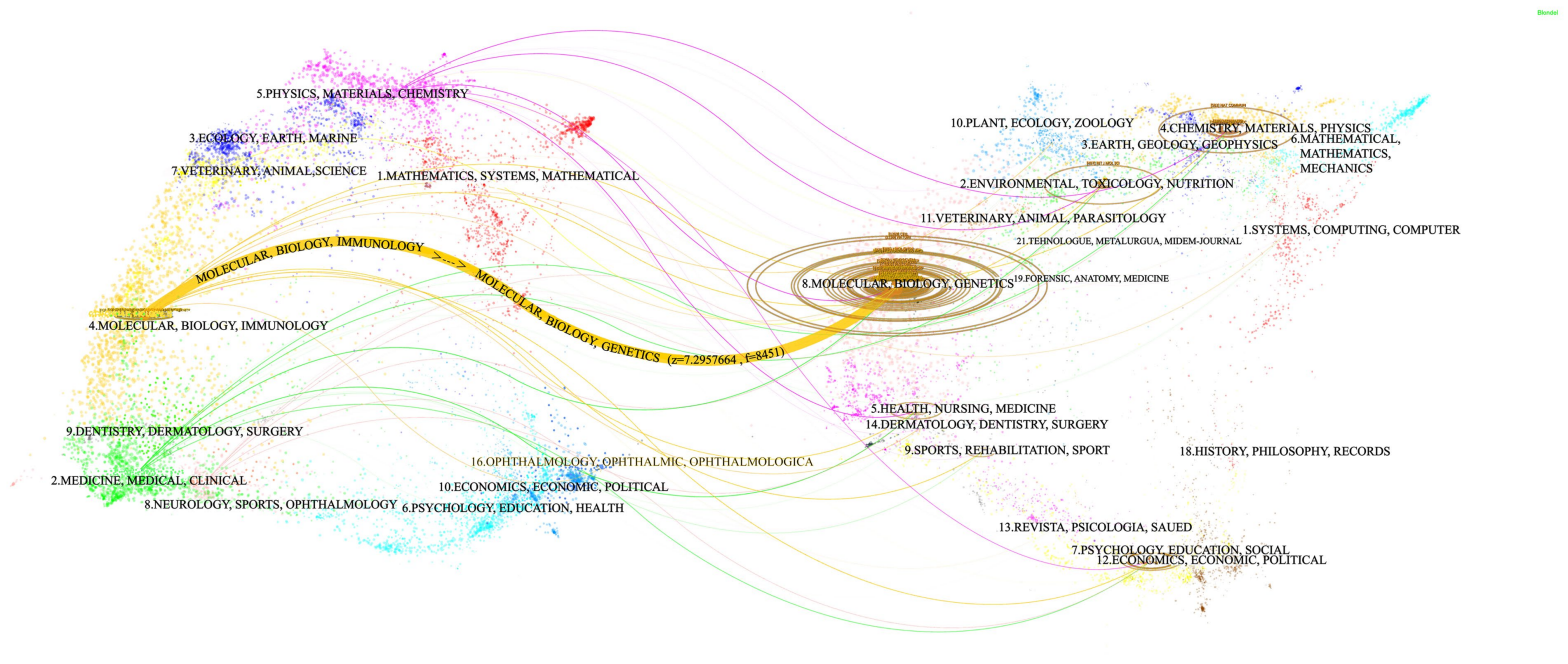
Journal double-figure overlay of ferroptosis in Parkinson’s disease.

The double graph overlay illustrates the citation relationships between journals and co-cited journals, with the citing journals clustered on the left and the co-cited journals clustered on the right. The curves represent citation paths, while the size of the ellipses reflects the NPs (Li et al., 2022). Using the *Z*-scores function in CiteSpace, a dual overlay visualization of the interaction between journals and their cited counterparts is created. Figure 5 reveals a prominent orange trajectory, indicating the primary citation pathway. This trajectory suggests that research in this domain is predominantly concentrated in molecular/biology/immunology journals, with citations largely originating from molecular/biology/genetics journals. This pattern underscores the interdisciplinary nature of research in ferroptosis and Parkinson’s disease, highlighting the engagement of experts from multiple disciplines. It also emphasizes the critical role of interdisciplinary communication and collaboration for advancing future research.

### 3.5 Authors and co-cited authors

**Figure 6:**
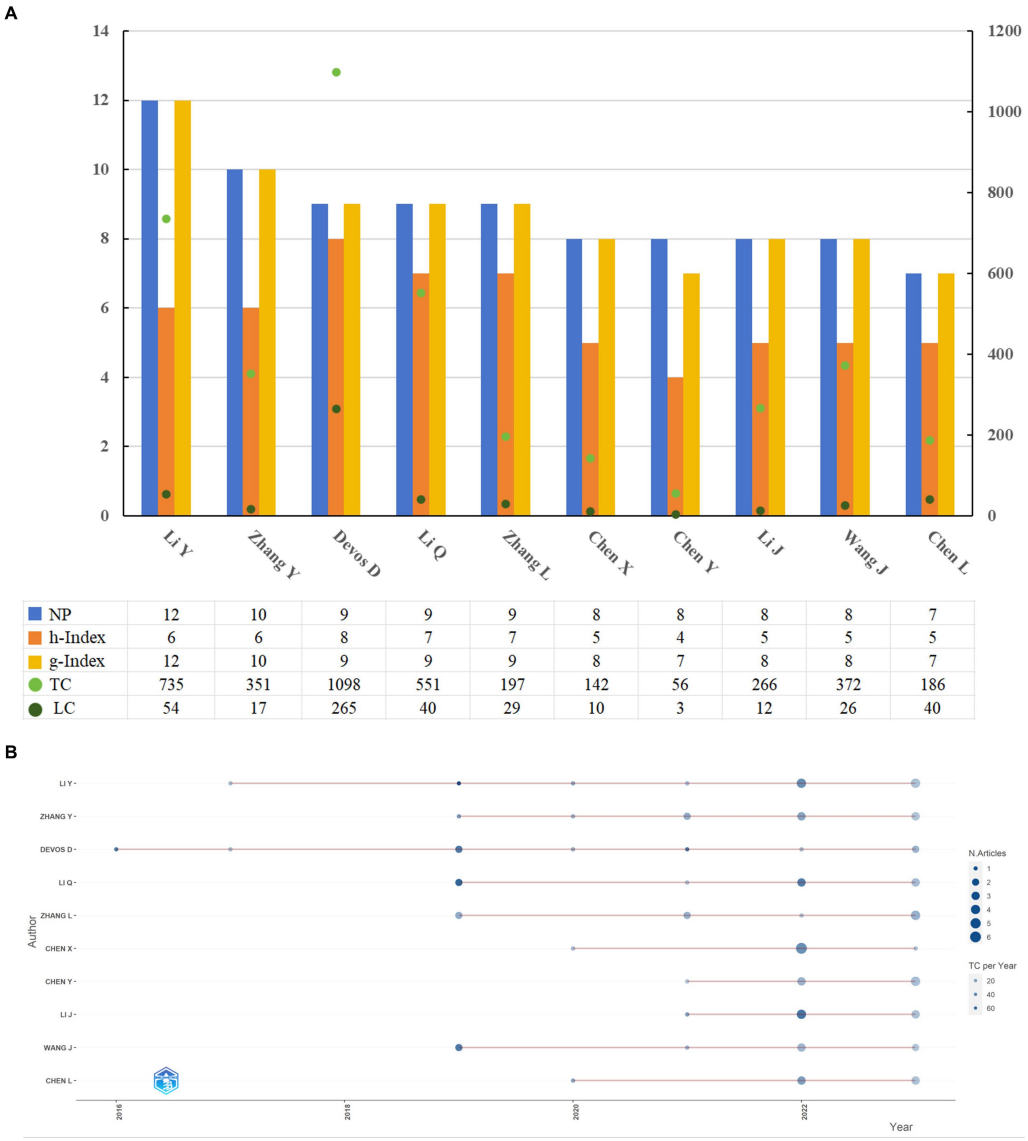
Relevant information displaying the 10 most productive authors **(A)**; the productivity of the 10 most productive authors over time **(B)**. NP, number of publications; TC, total citation; LC, local citation.

The study of ferroptosis in Parkinson’s disease involves contributions from 3,269 authors. The top 10 authors with the highest publication output are listed in Figure 6A, including their NPs, *h*-index, *g*-index, TCs, and local citations. Li Y. leads with the highest output (NP = 12, *h*-index = 6, *g*-index = 12), followed by Zhang Y. (NP = 10, *h*-index = 6, *g*-index = 10). Both authors have at least 10 publications in the field, but Li Y.’s higher *g*-index indicates a more substantial impact. Figure 6B illustrates the temporal evolution of publications and citations for these top 10 authors, showing a relationship between publication volume and citation frequency. Notably, Devos D. was an early contributor, publishing in 2016 with a paper titled “Ferroptosis, a newly characterized form of cell death in Parkinson’s disease that is regulated by PKC” (TC = 443). Li Y.’s 2019 publication, “Ischemia-induced ACSL4 activation contributes to ferroptosis-mediated tissue injury in intestinal ischemia/reperfusion,” received the highest citation count (TC = 470). In 2022, Chen X. had the highest annual publication count (NP = 6).

### 3.6 Co-occurrence of keywords

**Figure 7:**
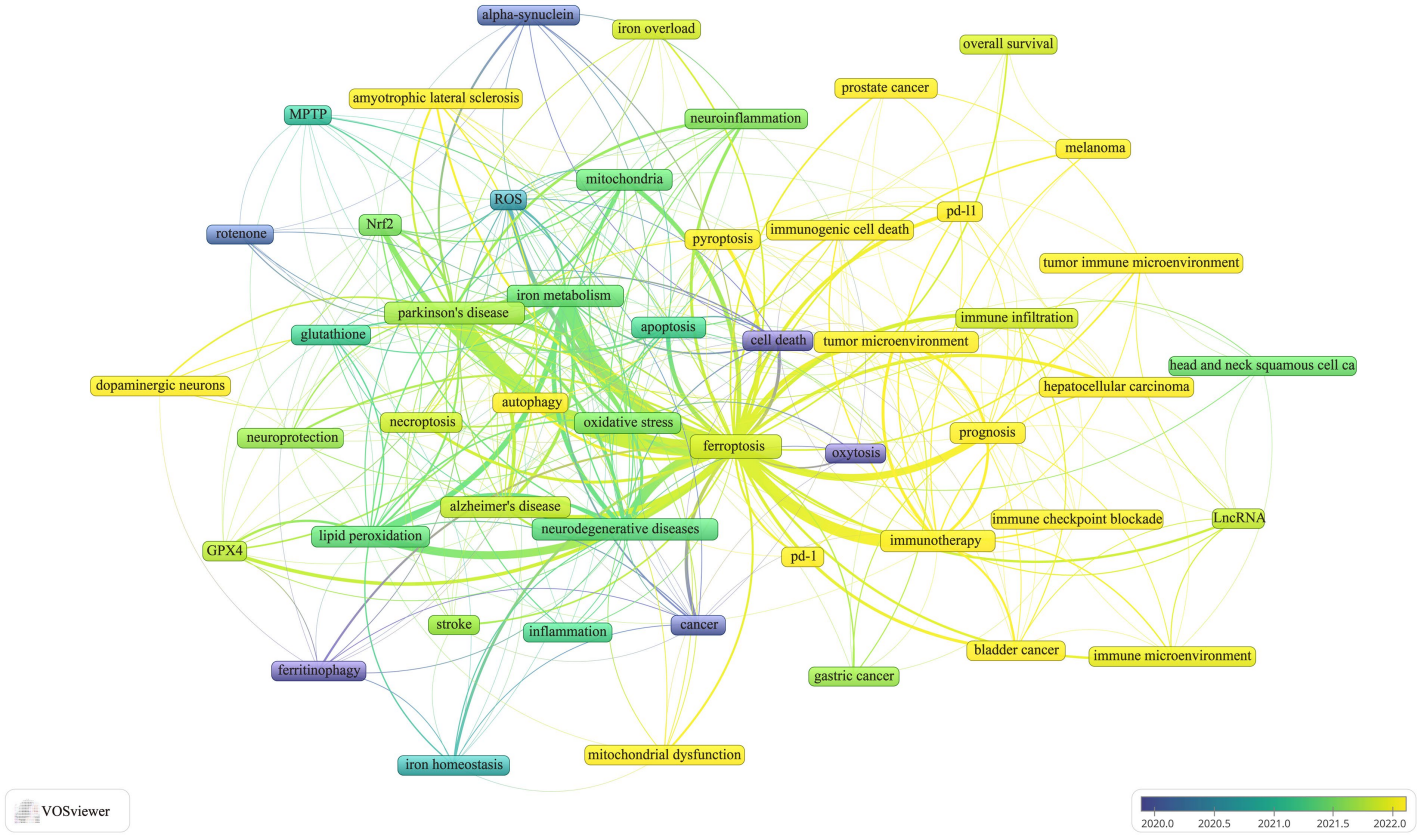
Keyword co-occurrence overlay visualization.

Keyword co-occurrence analysis is a powerful tool for identifying research hotspots and emerging trends. In this study, we analyzed a dataset of 1,053 author keywords using a threshold of five or more occurrences. This analysis, conducted with VOSviewer, resulted in 52 distinct keywords after consolidating synonyms. Figure 7 presents the visual analysis, where the thickness of the matrix lines denotes keyword frequency, and lines connecting nodes represent the strength of relationship between keywords. Thicker lines indicate more frequent co-occurrences within the same documents. Node colors differentiate clusters, with lighter shades highlighting dominant research directions. The analysis identified five main clusters, each representing a distinct research trajectory:

- Cluster 1: Keywords included ferroptosis, apoptosis, autophagy, necroptosis, pyroptosis, immunotherapy, immunogenic cell death (ICD), immune checkpoint blockade, gastric cancer, hepatocellular carcinoma, melanoma, tumor microenvironment, overall survival, PD-1, PD-L1, and prognosis.
- Cluster 2: Keywords included Parkinson’s disease, neurodegenerative diseases, alpha-synuclein, rotenone, MPTP, neuroprotection, neuroinflammation, inflammation, mitochondrial dysfunction, mitochondria, iron, Nrf2, oxeiptosis, and oxidative stress.
- Cluster 3: Keywords included Alzheimer’s disease, amyotrophic lateral sclerosis, cell death, lipid peroxidation, reactive oxygen species, glutathione, GPX4, iron metabolism, and iron overload.
- Cluster 4: Keywords included tumor immune microenvironment, immune microenvironment, immune infiltration, long non-coding RNAs (lncRNAs), bladder cancer, head and neck squamous cell carcinoma, and prostate cancer.
- Cluster 5: Keywords included cancer, dopaminergic neurons, stroke, ferrinophagy, and iron homeostasis.

Frequency analysis reveals that “immunotherapy,” “prognosis,” “tumor microenvironment,” and “mitochondrial dysfunction” are the current focal points of research. Among these, “immunotherapy” appears most frequently (*n* = 53), followed by “iron” (*n* = 48) and “neurodegenerative diseases” (*n* = 47). This indicates a significant interdisciplinary interest and convergence of research efforts on these themes.

**Figure 8:**
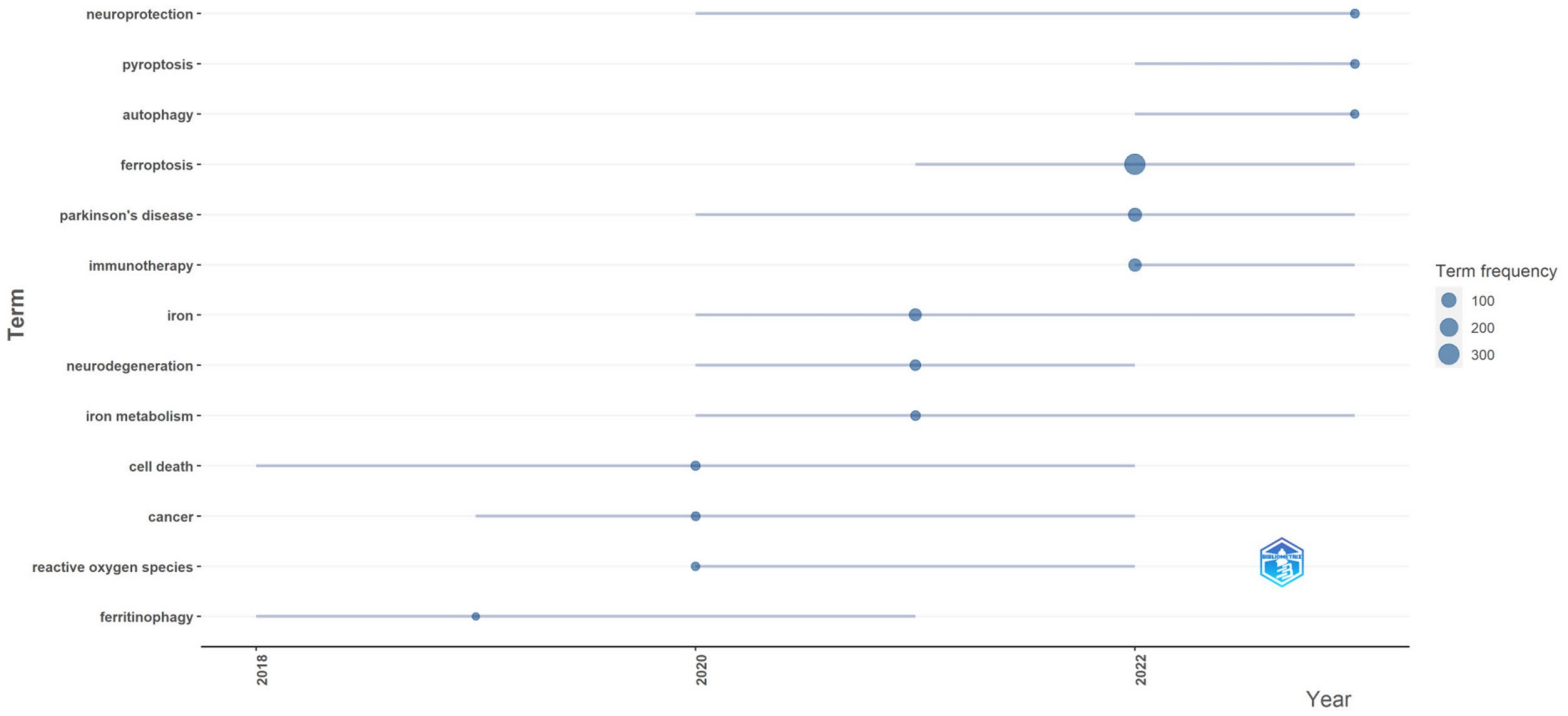
Keyword trend topic analysis.

Additionally, Figure 8 illustrates keyword trends from 2012 to 2023, filtering for terms with a frequency of five or more occurrences. The primary keywords during this period, besides “ferroptosis” and “Parkinson’s disease,” include “immunotherapy,” “prognosis,” “neuroprotection,” “pyroptosis,” and “autophagy.” These terms highlight the current main research directions, focusing on mechanisms of disease progression and potential therapeutic strategies (see Table 4).

**Table 4:** Top 22 keywords in the field of ferroptosis in Parkinson’s disease research.

Rank	Author keywords	Counts	Rank	Author keywords	Counts
1	Ferroptosis	309	12	Apoptosis	19
2	Parkinson’s disease	90	13	Iron metabolism	17
3	Immunotherapy	53	14	Mitochondria	17
4	Iron	48	15	PD-l1	16
5	Neurodegenerative diseases	47	16	Reactive oxygen species	16
6	Lipid peroxidation	36	17	Immune infiltration	15
7	Oxidative stress	36	18	Cell death	14
8	Prognosis	31	19	GPX4	14
9	Nrf2	21	20	Neuroinflammation	14
10	Tumor microenvironment	21	21	Hepatocellular carcinoma	13
11	Alzheimer’s disease	20	22	Cancer	12

### 3.7 Three-field plot of author-keyword-journal

**Figure 9:**
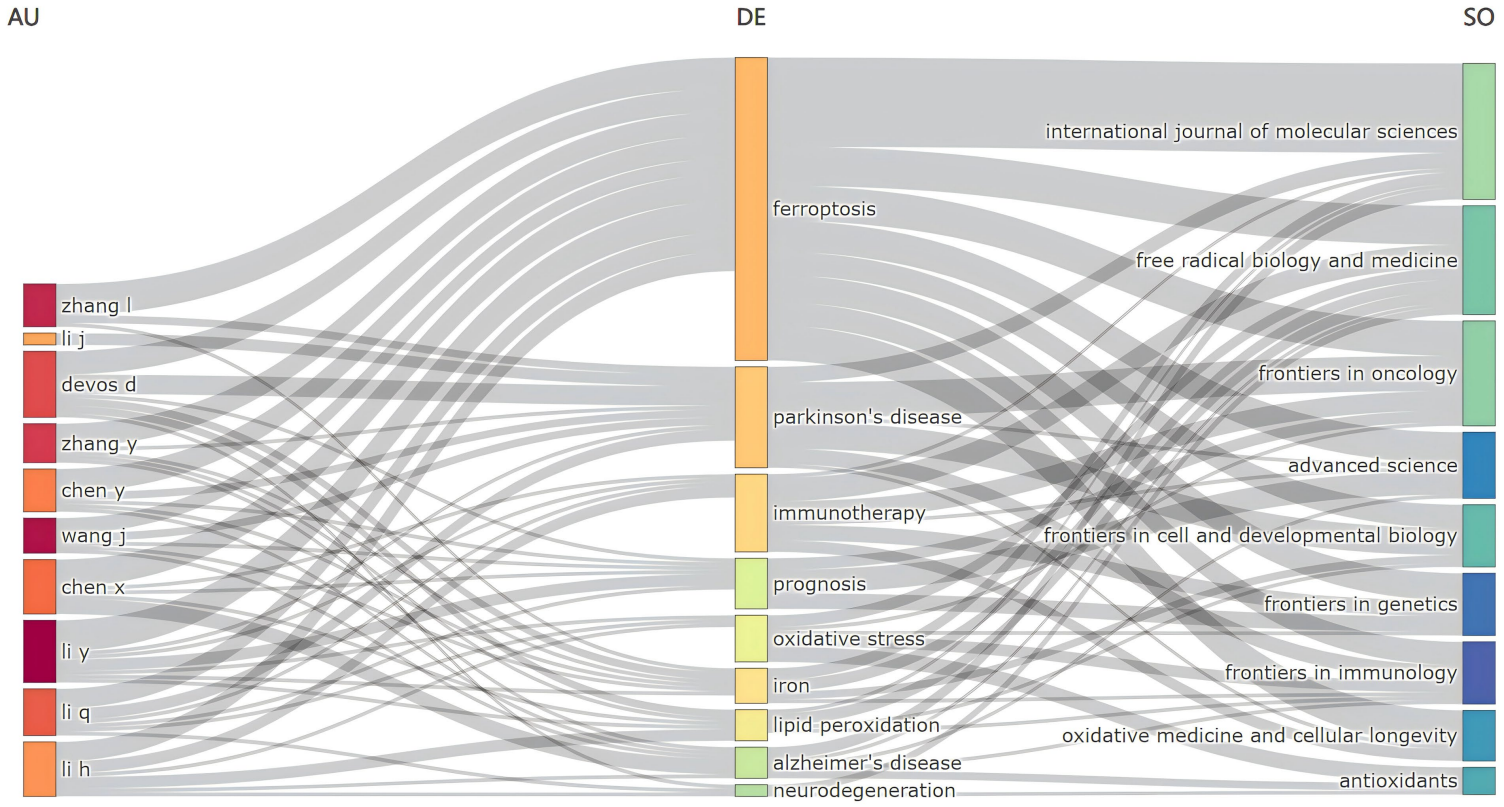
Three fields plot distribution of author-keyword-journal.

The three-field plot methodology enables a comprehensive synthesis and analysis of the relationships among various bibliometric indicators by constructing an intricate network map that links these indicators. Using the three-field plot approach within bibliometrix, we delineated three distinct domains: “author” on the left, “keyword” in the center, and “journal” on the right, resulting in the creation of an “author-keyword-journal” network, as shown in Figure 9. This figure highlights that, in addition to the central keywords “ferroptosis” and “Parkinson’s disease,” key research trajectories include “immunotherapy,” “prognosis,” “oxidative stress,” “lipid peroxidation,” and “tumor microenvironment.” A notable co-occurrence relationship is observed between authors and specific keywords. For example, Chen X. is strongly associated with “prognosis,” Li Y. with “prognosis,” and both Devedjian J. C. and Devos D. with “lipid peroxidation.” Furthermore, significant co-occurrence relationships are evident between keywords and specific journals: “ferroptosis” frequently appears in the *International Journal of Molecular Sciences*, “Parkinson’s disease” in *Free Radical Biology and Medicine*, “immunotherapy” in both *Frontiers in Cell and Developmental Biology* and *Frontiers in Oncology*, and “prognosis” in *Advanced Science* and *Frontiers in Genetics*. These findings highlight the influential authors and seminal contributions in the field and identify emerging research hotspots, underscoring the pivotal role of journals like the *International Journal of Molecular Sciences*, *Frontiers in Cell and Developmental Biology*, and *Free Radical Biology and Medicine* in shaping the research landscape.

### 3.8 Reference citation burst analysis

**Figure 10:**
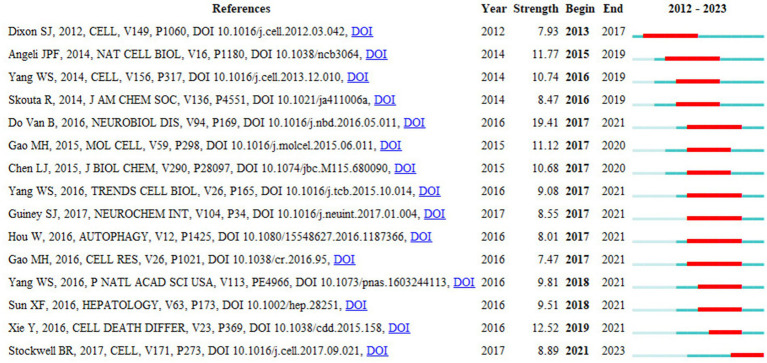
Citations burst graph of the first 15 references.

Reference citation burst analysis identifies literature that experiences a surge in citations within a specific period, reflecting research hotspots and trends. The blue line in Figure 10 indicates the time interval, while the red columns on the blue line represent burst periods. The strength of the burst reflects the importance of the literature in the research field, with “year” indicating the publication year, and “begin” and “end” marking the start and end of the citation burst. Using CiteSpace with parameters set to number of states = 2, γ[0, 1] = 1.0, and minimum duration = 2, we analyzed a total of 81 references. Among these, the reference with the strongest burst intensity was “Ferroptosis is a newly characterized form of cell death in Parkinson’s disease that is regulated by PKC by Do Van’s et al. (2016) study, indicating it is the most frequently cited. The earliest significant study in this field was “Ferroptosis: an iron-dependent form of nonapoptotic cell death” by Dixon et al. (2012). Additionally, “Ferroptosis: a regulated cell death nexus linking metabolism, redox biology, and disease” by Stockwell’s et al. (2017) is still experiencing a citation burst.

## 4 Discussion

### 4.1 Research status

Since the introduction of ferroptosis, research has increasingly highlighted its critical connection to a range of diseases, including neurodegenerative disorders, cancer, and ischemia-reperfusion injuries (Lee et al., 2020; Li et al., 2019). Despite this growing interest, bibliometric analyses specific to this field are still limited. This study provides a comprehensive bibliometric analysis of the relationship between ferroptosis and Parkinson’s disease, utilizing various bibliometric software tools. Our findings indicate that from 2012 to 2016, there were few publications addressing ferroptosis in Parkinson’s disease, reflecting an initial scarcity of research in this area. However, since 2017, there has been a notable increase in literature, with publications in this domain representing 58.09% of the total articles over the past 2 years. The volume of publications in 2023 was 1.7 times higher than that in 2022, underscoring ferroptosis in Parkinson’s disease as a rapidly growing research focus. This study aims to provide a reference for scholars entering this expanding field through bibliometric analysis and visual presentation.

Our analysis also reveals that China is at the forefront of publications in this field, with two-thirds of the top 12 research institutions based there. However, there is a significant lack of collaborative efforts both among Chinese institutions and with international partners. This limited collaboration could hinder the sustainable progress of research. To address this, enhancing academic exchanges and fostering international cooperation are crucial for advancing collective research on ferroptosis and Parkinson’s disease.

The journal analysis, based on Bradford’s law, identified 19 core journals within this field, collectively publishing 165 articles. Notably, nine journals from the Frontiers series accounted for 38.2% of these publications, spanning diverse areas such as neurology, oncology, genetics, and molecular biology. Among these, the *International Journal of Molecular Sciences* emerged as the leading venue, predominantly featuring articles on biosynthetic agents related to ferroptosis, including both inducers and inhibitors (Du and Guo, 2022). This journal extensively covers neurodegenerative disorders such as Parkinson’s disease, Alzheimer’s disease, and Huntington’s disease, focusing on the role of ferroptosis in disease mechanisms. This includes initiating cell death through lipid peroxidation, glutathione depletion, and iron accumulation, which can lead to inflammatory responses, myelin degeneration, astrocyte dysfunction, and cognitive impairments (Reichert et al., 2020). Additionally, the journal explores potential therapeutic targets for ferroptosis in neurological diseases (Peeples and Genaro-Mattos, 2022; Tang et al., 2023). *Free Radical Biology and Medicine*, known for its high citation count and *h*-index, specializes in examining oxidative reactions’ roles in diseases, including the mechanisms of ferroptosis and associated treatment strategies. Research published in this journal has significantly advanced the understanding of ferroptosis and its relationship with Parkinson’s disease (Anandhan et al., 2022; Xia et al., 2024; Zhang et al., 2020).

Most publications in this research area are concentrated in molecular biology, immunology, and genetics, with comparatively fewer articles in clinically oriented journals. This suggests that the application of ferroptosis research to clinical practice for Parkinson’s disease remains underdeveloped. There is a critical need for increased interdisciplinary collaboration to bridge the gap between fundamental research and clinical applications.

A visual analysis of author publication outputs revealed that 90% of the top 10 authors in this field are affiliated with institutions in China. Conrad M is noted for having the highest citation count for published articles, followed by Bush A. I., Friedmann Angeli J. P., Bayir H., and Kagan V. E. This trend highlights the significant contributions of Chinese scholars to the field. However, there is a need to enhance the quality of publications and to promote more rigorous academic discourse. Fostering collaboration among researchers will be essential for advancing more profound and nuanced investigations in this domain.

Reference analysis indicates that since the introduction of the ferroptosis concept, various ferroptosis inducers (such as erastin, sorafenib, and buthionine sulfoximine) and inhibitors (such as iron chelators, Fer-1 derivatives, PKC inhibitors) have been explored (Do Van et al., 2016). There is increasing anticipation that ferroptosis inhibitors may serve as adjunctive therapies for neurodegenerative disorders, including Parkinson’s disease (Zhang et al., 2023). Research has demonstrated that iron accumulation in the brain establishes a complex relationship between ferroptosis and Parkinson’s disease (Lin et al., 2022). Additionally, GPX4, a critical antioxidant enzyme in mammals, plays a pivotal role in the ferroptosis pathway and in therapeutic interventions for the disease (Liu et al., 2023; Ursini and Maiorino, 2020).

### 4.2 Research hot spots and frontiers

The keyword analysis, depicted in Figure 7, highlights Cluster 1 as the predominant research focus, which includes ferroptosis, apoptosis, autophagy, necroptosis, pyroptosis, immunotherapy, tumor microenvironment, immune checkpoint inhibitors, and ICD. Emerging evidence suggests that therapies targeting non-apoptotic cell death pathways—specifically ferroptosis, pyroptosis, and autophagy—could potentially enhance the effectiveness of immunotherapy (Tang et al., 2020). Ferroptosis, necroptosis, and pyroptosis are increasingly recognized as novel forms of ICD that may influence the tumor microenvironment. Combining inducers of these cell death pathways with immune checkpoint inhibitors could amplify tumor immunotherapy outcomes (Gao et al., 2022; Niu et al., 2022).

Research also highlights that factors such as ferroptosis, apoptosis, autophagy, necrosis, oxidative stress, neuromelanin, and neuroinflammation are integral to neurodegenerative processes (Chen et al., 2020; Moreno-Garcia et al., 2021; Pomilio et al., 2023; Silva et al., 2022). Future research should focus on the sequential development of various cell death modalities in Parkinson’s disease models to identify stage-specific interventions that may slow or halt disease progression.

Furthermore, the co-occurrence analysis of keywords reveals a significant relationship between lncRNAs, ferroptosis, immunotherapy, and the tumor microenvironment. Recent studies have identified several lncRNAs related to ferroptosis that play crucial roles in disease progression. For instance, lncRNA NEAT1 regulates ferroptosis sensitivity in non-small-cell lung cancer by interacting with ACSL4 and GPX4 (Wu and Liu, 2021). Additionally, lncRNA NEAT1 impacts Parkinson’s disease pathology by sponging miR-212-3p, which targets AXIN1, while lncRNA SNHG1 exacerbates neuroinflammation in Parkinson’s disease through the miR-7/NLRP3 pathway (Cao et al., 2018; Liu et al., 2021). Given that ACSL4 and GPX4 are pivotal regulators of ferroptosis, lncRNA NEAT1 might modulate ferroptosis via these molecules, presenting a promising therapeutic approach for Parkinson’s disease (Liu et al., 2021; Zhou et al., 2023). Future studies should aim to elucidate the precise mechanisms by which NEAT1 affects ferroptosis and neurodegenerative diseases, and explore targeted therapies that modulate this lncRNA.

In addition to the aforementioned keywords, oxidative stress emerges as a critical term in this field. Oxidative stress is characterized by an imbalance between pro-oxidants (e.g., free radicals) and antioxidants, leading to excessive reactive oxygen species (Dossena and Marino, 2021; Sies, 2015). This imbalance damages cellular components, including DNA, proteins, and lipids (Martinez and Kannan, 2018). Oxidative stress is a major factor in the pathogenesis of Parkinson’s disease and other neurodegenerative disorders, and is a key feature of ferroptosis (Barnham et al., 2004; Dionisio et al., 2021; Yu et al., 2021). Therefore, future research should focus on a detailed examination of the relationship between ferroptosis and Parkinson’s disease, with a particular emphasis on the role of oxidative stress in this relationship.

### 4.3 Advantages and disadvantages

This study offers several distinct advantages. First, bibliometric analyses focused on the intersection of ferroptosis and Parkinson’s disease are scarce, making this investigation a pioneering effort. By using a range of bibliometric tools, this study systematically analyzes the existing body of research, providing an objective and thorough examination of the field. This approach yields valuable insights and serves as a significant reference for scholars exploring this niche area. Second, bibliometric analysis surpasses traditional research methods by offering a more expansive view of research hotspots and emerging trends, thus facilitating a deeper understanding of both current dynamics and future directions in the field.

Nevertheless, the study has some limitations too. First, it relies solely on data from the WoSCC database, which may exclude relevant studies indexed in other databases. Second, focusing exclusively on English-language publications could result in an underrepresentation of research published in other languages. Furthermore, newly published studies, especially those from the current year that have yet to achieve significant citation counts, may be underrepresented. To address this, continuous monitoring of emerging research is essential. Despite these limitations, the literature analyzed provides a comprehensive overview of the current state of research, identifying key trends and future research directions in the study of ferroptosis in Parkinson’s disease.

## 5 Conclusion

The volume of scholarly work exploring the relationship between ferroptosis and Parkinson’s disease has shown a notable upward trend, with China emerging as a prominent contributor to this research domain. Collaborative efforts across institutions, countries, and researchers have significantly advanced understanding in this field. The focus of research has shifted over time from foundational studies on the mechanisms of ferroptosis to more applied clinical evaluations concerning its implications for treatment and prognosis in ferroptosis-related diseases. Ferroptosis has been identified as a crucial regulatory factor in a range of diseases, with recent findings underscoring its significant role in the pathogenesis of neurodegenerative conditions, particularly Parkinson’s disease. This evolving landscape highlights ferroptosis as a critical area of study and suggests that it will continue to be a central focus for future research. Continued interdisciplinary collaboration and research are essential to further unravel the complexities of ferroptosis and its impact on disease mechanisms and treatments.

## Data Availability

The original contributions presented in the study are included in the article/Supplementary material, further inquiries can be directed to the corresponding author.
