# Supplementary material for: Knowledge mapping of ferroptosis in Parkinson’s disease: a bibliometric analysis: 2012–2023
Source: Front Aging Neurosci. 2024 Aug 30;16:1433325. doi: 10.3389/fnagi.2024.1433325 (PMC11401074; doi:10.3389/fnagi.2024.1433325)
Supplement: Supplementary file 1 [file Table_1.DOCX]

Supplementary Material

# Supplementary Figures and Tables

Table 5: Summary of the main studies of the 15 references in the order of their references in Figure 10

| Rank | Burst Strength | First author/year of publication | Source Journal | Main Content |
| --- | --- | --- | --- | --- |
| 1 | 7.93 | Dixon SJ/2012 | Cell | Erastin induces a form of cell death that is morphologically, biochemically, and genetically distinct from apoptosis, necrosis, and autophagy through Systemxc-inhibition of cystine uptake, named Ferroptosis; and identifies Ferrodostatin-1 as a potent ferroptosis inhibitor |
| 2 | 11.77 | Angeli JPF/2014 | Nature cell biology | This study focuses on GPX4 as a pivotal antioxidant enzyme in mammals, particularly highlighting its critical function in inhibiting lipid peroxidation and preventing ferroptosis. Research findings indicate that a deficiency in GPX4 results in augmented lipid peroxidation, potentially culminating in ferroptosis. Additionally, the application of iron chelators has been demonstrated to mitigate cell death attributable to GPX4 depletion. |
| 3 | 10.74 | Yang WS/2014 | Cell | The authors used metabolomic analysis to analyze that Erastin inactivates glutathione peroxidases (GPXs) by depleting glutathione, and used chemical proteomics to find that RSL3 directly binds to GPX4 to inactivate GPX4; the study also proved that through Inducing ferroptosis can inhibit tumor growth in animal models, indicating that GPX4 is an important regulator of ferroptosis in cancer cells. |
| 4 | 8.47 | Skouta R/2014 | Journal of the American Chemical Society | This study found that Ferrostatin-1 (Fer-1), as an inhibitor of ferroptosis, can inhibit cell death in various disease models (Huntington's disease, per cerebral leukomalacia, and renal dysfunction) by inhibiting lipid peroxidation, but it does not affect the production of reactive oxygen species by mitochondria or the permeability of lysosomal membranes. The study further explored the mechanism of action of Fer-1 through structure-activity relationships (SAR) and discovered more potent compounds Ferrostatins, which may form the basis of future drugs for the treatment of tissue damage mediated by lipid peroxidation. |
| 5 | 19.41 | Do Van B/2016 | Neurobiology Of Disease | The study found that activation of PKCα may trigger ferroptosis and activate MEK in an RAS-independent manner; this study is the first to highlight the importance of ferroptosis in Parkinson's disease. In neurodegenerative diseases (such as PD), iron chelators, Fer-1 derivatives, and PKC inhibitors may serve as drug candidates to prevent neuronal loss. |
| 6 | 11.12 | Gao MH/2015 | Molecular Cell | Using mouse embryonic fibroblasts (MEFs) to establish different cell models, the study found that the transferrin receptor on the cell surface and the intracellular glutamine cleavage pathway are key components of ferroptosis; inhibiting glutamine cleavage, Organ damage induced by ischemia-reperfusion can be treated. |
| 7 | 10.68 | Chen LJ/2015 | Journal Of Biological Chemistry | Using the Gpx4 neuron inducible knockout (Gpx4NIKO) mouse model, it was found that knocking out Gpx4 can lead to ferroptosis, and knocking out Gpx4 can lead to degeneration of spinal motor neurons, but has no obvious effect on cerebral cortex neurons; deletion of Gpx4 Caused motor neuron degeneration characterized by ferroptosis, thus proving that inhibiting ferroptosis is of great significance to motor neurons, providing a new perspective for understanding motor neuron degeneration (such as amyotrophic lateral sclerosis), and providing a new perspective for the future treatment strategies provide new targets. |
| 8 | 9.08 | Yang WS/2016 | Trends in Cell Biology | Ferroptosis is a regulated cell death driven by the loss of GPX4 activity and the accumulation of lipid reactive oxygen species, especially lipid peroxides. The authors of this article summarize the discovery, inducers, inhibitors, regulatory mechanisms, and role of ferroptosis in normal physiology and pathophysiology. |
| 9 | 8.55 | Guiney SJ/2017 | Neurochemistry International | This article mainly discusses the role of ferroptosis and other cell death pathways in Parkinson's disease and how these cell death pathways can be therapeutic targets to slow disease progression. Treatment methods such as iron chelators and glutathione precursor N-acetylcysteine ​​(NAC) have shown potential in the treatment of PD. It shows that forebrain neurons are sensitive to ferroptosis, suggesting that ferroptosis may be the main mechanism leading to neuronal degeneration in PD. |
| 10 | 8.01 | Hou W/2016 | Autophagy | Studies have found that in fibroblasts and cancer cells, autophagy promotes ferroptosis by degrading ferritin. Gene knockout or silencing of Atg5 and Atg7 inhibits Erastin-induced ferroptosis. In ferroptosis, NCOA4 is a selective transport receptor for ferritin-selective autophagy. Likewise, genetic inhibition of NCOA4 inhibits ferritin degradation and suppresses ferroptosis. These findings provide new insights into the interplay between autophagy and regulated cell death. |
| 11 | 7.47 | Gao MH/2016 | Cell Research | Multiple autophagy-related genes were discovered as positive regulators of ferroptosis through RNAi screening and genetic analysis. The occurrence of ferroptosis results in the activation of autophagy and degradation of ferritin and the ferritin autophagy transport receptor NCOA4. Inhibiting ferritin autophagy by blocking autophagy or knocking out NCOA4 removes intracellular free iron and reactive oxygen species, ultimately inhibiting ferroptosis. Ferroptosis is therefore an autophagic cell death process. |
| 12 | 9.81 | Yang WS/2016 | Proceedings Of The National Academy Of Sciences Of The United States Of America | Lipoxygenase was found to be required for ferroptosis by oxidizing polyunsaturated fatty acids (PUFAs) (particularly the diallyl position) through a phosphorylated kinase G2 (PHKG2)-dependent iron pool, using deuterated PUFAs (D -PUFAs) treatment of cells can block the oxidation of PUFA and inhibit ferroptosis. The study further revealed that ferroptosis inducers inhibit GPX4 by covalently targeting the active site selenocysteine ​​of GPX4, leading to the accumulation of PUFA superoxides, thereby inducing ferroptosis in cells, which can be eliminated by eliminating PUFA superoxides. Inhibiting cellular ferroptosis and proposing new strategies to control ferroptosis. |
| 13 | 9.51 | Sun XF/2016 | Hepatology | Using cell and animal models, the authors found that activating the p62-Keap1NRF2 pathway inhibited ferroptosis in HCC cells. When hepatoma cells were exposed to ferroptosis-inducing compounds (such as erastin, sorafenib, and buthionine sulfoximine), the expression of p62 blocked NRF2. degradation, and enhanced NRF2 nuclear accumulation through inactivation of Keap-1. In addition, nuclear NRF2 interacts with the transcriptional coactivator small V-Maf musculoaponeurotic fibrosarcoma oncogene homologous recombination protein (such as MafG), which then activates the transcription of NQO1, HO-1, and FTH1. Knockdown of p62, NQO1, HO1, and FTH1 by RNA interference in hepatocellular carcinoma cells promoted the ferroptosis response to elastin and sorafenib. In addition, genetic or pharmacological inhibition of NRF2 expression in hepatocellular carcinoma cells increased the anticancer activity of erastin and sorafenib. This study found that the status of NRF2 determines the response of HCC cells to ferroptosis, both in vitro and in tumor xenograft models. Key factors in response to targeted therapies. |
| 14 | 12.52 | Xie Y/2016 | Cell Death & Differentiation | This article elaborates on the discovery, morphological characteristics, inducers, inhibitors, involved signaling pathways, molecular biology mechanisms, detection methods, etc. of ferroptosis, and discusses the role of ferroptosis in disease. effect. |
| 15 | 8.89 | Stockwell BR/2017 | Cell | This article explains the basic mechanism of ferroptosis. The sensitivity of ferroptosis is related to various biological metabolic processes such as amino acid, iron, polyunsaturated fatty acid metabolism, etc., and has been found in neurodegenerative diseases such as Alzheimer's disease, Huntington's disease, and Parkinson's disease. Ferroptosis is found in diseases. In addition, ferroptosis may have tumor-suppressive functions and can be used as a potential means of cancer treatment. The article also explores the connections between ferroptosis and metabolism, oxidative biology, and disease, highlights connections to other areas of biology and medicine, and recommends tools and guidelines for studying this novel type of regulated cell death. |
